# Disease Progression of Hypertrophic Cardiomyopathy: Modeling Using Machine Learning

**DOI:** 10.2196/30483

**Published:** 2022-02-02

**Authors:** Matej Pičulin, Tim Smole, Bojan Žunkovič, Enja Kokalj, Marko Robnik-Šikonja, Matjaž Kukar, Dimitrios I Fotiadis, Vasileios C Pezoulas, Nikolaos S Tachos, Fausto Barlocco, Francesco Mazzarotto, Dejana Popović, Lars S Maier, Lazar Velicki, Iacopo Olivotto, Guy A MacGowan, Djordje G Jakovljević, Nenad Filipović, Zoran Bosnić

**Affiliations:** 1 Faculty of Computer and Information Science University of Ljubljana Ljubljana Slovenia; 2 Unit of Medical Technology and Intelligent Information Systems Department of Materials Science and Engineering University of Ioannina Ioannina Greece; 3 Department of Experimental and Clinical Medicine University of Florence Florence Italy; 4 Cardiomyopathy Unit Careggi University Hospital University of Florence Florence Italy; 5 National Heart and Lung Institute Imperial College London London United Kingdom; 6 Clinic for Cardiology Clinical Center of Serbia University of Belgrade Belgrade Serbia; 7 Department of Internal Medicine II (Cardiology, Pneumology, Intensive Care Medicine) University Hospital Regensburg Regensburg Germany; 8 Faculty of Medicine University of Novi Sad Novi Sad Serbia; 9 Institute of Cardiovascular Diseases Vojvodina Sremska Kamenica Serbia; 10 Translational and Clinical Research Institute Faculty of Medical Sciences Newcastle University Newcastle upon Tyne United Kingdom; 11 Faculty of Health and Life Sciences Coventry University Coventry United Kingdom; 12 Bioengineering Research and Development Center Kragujevac Serbia

**Keywords:** hypertrophic cardiomyopathy, disease progression, machine learning, artificial intelligence, AI, ML, cardiomyopathy, cardiovascular disease, sudden cardiac death, SCD, prediction, prediction model, validation

## Abstract

**Background:**

Cardiovascular disorders in general are responsible for 30% of deaths worldwide. Among them, hypertrophic cardiomyopathy (HCM) is a genetic cardiac disease that is present in about 1 of 500 young adults and can cause sudden cardiac death (SCD).

**Objective:**

Although the current state-of-the-art methods model the risk of SCD for patients, to the best of our knowledge, no methods are available for modeling the patient's clinical status up to 10 years ahead. In this paper, we propose a novel machine learning (ML)-based tool for predicting disease progression for patients diagnosed with HCM in terms of adverse remodeling of the heart during a 10-year period.

**Methods:**

The method consisted of 6 predictive regression models that independently predict future values of 6 clinical characteristics: left atrial size, left atrial volume, left ventricular ejection fraction, New York Heart Association functional classification, left ventricular internal diastolic diameter, and left ventricular internal systolic diameter. We supplemented each prediction with the explanation that is generated using the Shapley additive explanation method.

**Results:**

The final experiments showed that predictive error is lower on 5 of the 6 constructed models in comparison to experts (on average, by 0.34) or a consortium of experts (on average, by 0.22). The experiments revealed that semisupervised learning and the artificial data from virtual patients help improve predictive accuracies. The best-performing random forest model improved R^2^ from 0.3 to 0.6.

**Conclusions:**

By engaging medical experts to provide interpretation and validation of the results, we determined the models' favorable performance compared to the performance of experts for 5 of 6 targets.

## Introduction

### Background

Recent reviews of machine learning (ML) applications in cardiovascular medicine [1,2] suggest that the use of ML is on the rise and that it is being adopted by doctors in their daily practice. ML applications in cardiology are reflected by augmenting medical practice by contributing to early diagnosis, risk stratification, and personalized therapeutics. The examples of such applications in other domains include modeling disease progression of Alzheimer disease [3,4], Parkinson disease [5], multiple sclerosis [6], chronic kidney disease [7], chronic liver disease [8], and others.

Cardiovascular disorders in general are responsible for 30% of deaths worldwide. Among them specifically, hypertrophic cardiomyopathy (HCM) is a genetic cardiac disease that is a cause of sudden cardiac death (SCD), especially among young adults and athletes [9]. Cardiovascular diseases represent groups of diseases that can greatly benefit from preemptive prediction, prevention, and proactive management; thus, this opens an opportunity for methods of artificial intelligence (AI) [2]. Disease progression is especially hard to detect in slow-progressing diseases, such as HCM, which is present in about 1 of 500 young adults [10]. Although HCM has 4 identified stages [11], patients with HCM can experience a sudden cardiac arrest or the disease can slowly progress over several years. Currently, the state-of-the-art HCM Risk-SCD calculator method for risk stratification of patients diagnosed with HCM [12] is widely used in practice. Although this method predicts the risk of SCD, no methods, to the best of our knowledge, are available for modeling the patient's clinical status up to 10 years ahead. Detection of cardiovascular risk for 10 years ahead is important and has been recently modeled for atherosclerotic cardiovascular disease [13].

In this paper, we propose a novel ML-based tool for predicting disease progression for patients diagnosed with HCM in terms of adverse remodeling of the heart during a 10-year period. The method consists of 6 contemporaneous predictive regression models that independently predict future values of the following 6 clinical characteristics: left atrial diameter (LA_d), left atrial volume (LA_Vol), left ventricular ejection fraction (LVEF), New York Heart Association (NYHA) functional classification, left ventricular internal diameter at end diastole (LVIDd), and left ventricular internal diameter at end systole (LVIDs). Each prediction is supplemented with the explanation that is generated using the Shapley additive explanation (SHAP) method [14]. Comparison between current and future values of these 6 parameters, as well as the interpretation of the change, generated by explanation methods, can help cardiologists gain insight into the disease progression trend for a given patient.

### Machine Learning Methods in Medicine

ML techniques are being frequently applied in medicine to improve the prediction of disease progression, extraction of medical knowledge for outcome research, therapy planning and support, and overall patient management [15]. A wide variety of ML approaches can solve challenging problems in these tasks. For example, diseases such as Alzheimer disease, diabetes, and chronic obstructive pulmonary disease (COPD) progress slowly over the years. For modeling of COPD, a Markov model was proposed by Wang et al [16], who also included a database of virtual patients. Their method successfully modeled progression trajectories, showing that multiple progression trajectories are possible for some diseases.

In cardiology, there are several works addressing disease progression trends related to different cardiological diseases. With the increase in computational power, ML has become a tool to analyze nonlinear dependencies that are present either in relational data or in images. Juarez-Orozco et al [17] emphasized the advantages of ML, especially deep learning, in cardiac nuclear imaging, where ML can aid with ischemia diagnosis and event prognosis. Sardar et al [18] emphasized the advantages of AI in interventional cardiology, which is promising to bring a paradigm shift in the practice of medicine by improving real-time clinical decision making and standardizing robotic medical procedures. While focusing on the use of ML in electrocardiogram (ECG) analysis, Elul et al [19] also stated the crucial disadvantages of ML, which include a lack of explanation, relating the automated diagnosis with medical knowledge, and transparency of the system’s limitations. In their work, the authors proceeded to flag individual predictions that are irrelevant or not useful. To summarize, the mentioned works characterize AI as a developing tool that, with the synergy between humans and machines, will help transform medical practice and clinical care.

Further, a hybrid approach for progression of Parkinson disease [5] was successfully used by combining a variety of ML methods from different families: clustering, dimensional reduction, and incremental support vector regression. Deep learning was used for predicting Alzheimer disease, on average, about 6 years in advance [20] and for modeling Alzheimer disease progression [4]. Conditional restricted Boltzmann machines were also used for prediction of disease progression [3]. The authors simulated patient trajectories using 18 months of longitudinal data of around 1900 patients and showed that patient-level simulations are feasible using ML and appropriate data.

Several other ML approaches also model disease progression well in other medical domains, such as kidney disease progression [7]. In this work, 9 ML approaches were tested: linear regression (LR), elastic net regression, lasso regression, ridge regression, support vector machines (SVMs), random forests (RFs), k-nearest neighbors (KNNs), neural networks (NNs), and XGBoost. Similarly, ML models were applied to the problem of disease progression for hepatitis C virus [8] for the 5-year prediction problem using longitudinal data. The authors' conclusion was that the boosted survival tree-based models using longitudinal data perform better than cross-sectional or linear models. Last but not least, ML was also used for disease progression and secondary progression detection for multiple sclerosis [6]. Several ML models were evaluated for predictions of disease severity in 6-10 years, such as KNNs, decision trees, LR, and SVMs. SVMs performed best.

To summarize, the overview indicates that ML models can be successfully applied to problems of predicting disease progression, which is also the goal of this paper. In the next subsection, we overview how ML approaches are used in cardiology, specifically for HCM, which is the focus of this paper.

### Machine Learning for Modeling Hypertrophic Cardiomyopathy

Most ML contributions to cardiovascular medicine focus on risk stratification of patients. One of the biggest obstacles to using data for a broader variety of ML applications is that data are usually stored in diverse repositories, which are not readily usable for cardiovascular research, due to various data quality challenges [2]. Where the data are readily available, different ML algorithms have been successfully used, such as Wasserstein generative adversarial networks [21], convolutional NNs [22,23], deep NNs [24], and boosted decision trees [25]. Some authors have tested multiple models, such as RFs, artificial NNs, SVMs, and Bayesian networks [26], or a combination of J48, naive Bayes, KNNs, SVMs, RFs, bagging, and boosting [27]. Cuocolo et al [1] overviewed ML methods in cardiology, emphasizing their successful applications for building clinical predictive models, for analyzing ECG signals and image data. For the latter problems, the most successful methods were NNs, deep NNs, and convolutional networks. Advances in prediction accuracy have also been made by using deep NNs to make predictions based on fast, large-scale genome-wide association studies [28].

HCM is a severe disease for which 4 stages of progression have been identified in the medical literature [11]. Current state-of-the-art ML mostly uses only statistical models, such as multivariate regression analysis, which uses preselected predictor variables of known medical importance. Cardiac magnetic resonance (CMR) images [29,30] and echocardiographic diagnostics [31] are found to be a good source of important attributes for HCM identification. Recently, researchers have started proposing ML-based risk stratification for patients diagnosed with comorbidities to separate patients into low- and high-risk categories or several categories on a scale [32]. The medical literature is mostly focused on finding risk factors that identify increased risk of SCD in patients with HCM [12,33]. A study [34] presenting the guidelines used in risk stratification for patients with HCM proposed potential SCD modifiers. Maron et al [35] performed a similar study on older populations and also summarized risk factors that could prevent SCD. The continuation of this research [36] aimed to develop an accurate strategy to assess the reliability of SCD prediction methods in prevention of SCD in patients diagnosed with HCM.

It is important to note that patients with HCM who experience cardiac arrest are not identified by typical risk markers used in the American College of Cardiology or the statistical mathematical risk model by the European Society of Cardiology [37]. Therefore, new risk factors have been and still need to be considered and developed to provide additional information to better assess HCM risk. In our work, we focus on modeling the future development of HCM by predicting the change in relevant cardiac parameters 10 years ahead.

### Aims and Contributions

Novelties and contributions of this paper include:

- A disease progression system that comprises models for prediction of 6 contemporaneous relevant clinical parameters that are relevant to HCM for 10 years ahead. The system includes the implementation of the explanation methodology that provides interpretability of predictive models.
- Analysis of predictive performance if training data are extended using semisupervised learning or with artificial patient data.
- Validation of predictive accuracy with medical experts by comparing ML and human accuracy and by analyzing sensibility of the computer-generated prediction explanations.

The aim of this paper is to develop a system capable of detecting slow progression of HCM based on longitudinal data.

## Methods

### Modeling Disease Progression

**Figure 1:**
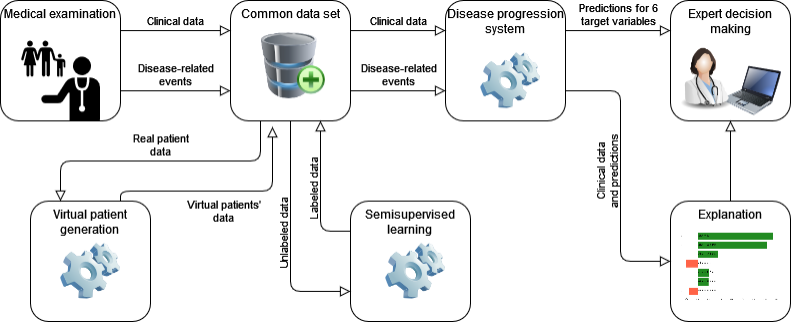
Overview of the proposed disease progression system. The system receives clinical data and disease-related events of a patient as input, uses virtual patient data and semisupervised learning for self-improvement, and returns the predictions and their explanation for 6 target variables.

In this work, we modeled disease progression by predicting 6 relevant patient parameters 10 years in advance. These parameters are indicators of HCM and can be used to determine the stage of HCM according to the known guidelines [37]. Additionally, a preliminary analysis was performed to verify the prediction strength of the chosen parameters, validating our choice, as described in the Data Set section. The proposed disease progression system (Figure 1) takes as input patients’ clinical data and data about their past disease-related events, such as dates of atrial fibrillation or syncope.

The output of the system is a set of 6 contemporaneous target predictions for parameters:

- LA_d
- LA_Vol
- LVEF
- LVIDd
- LVIDs
- NYHA functional classification

In addition to predictions, the system also generates their explanations, revealing the factors with the largest impact on the increase or decrease in the 6 target variables throughout the 10-year period.

We trained the proposed disease progression system using supervised ML techniques. To further improve the results, we augmented the original data using unlabeled data (semisupervised learning) and virtual patients’ data. We applied the semisupervised learning using patients without 10-year follow-ups and generated virtual patients’ data using various techniques for artificial data generation. The semisupervised learning first predicted patients' targets using the trained models on labeled data, so they could be afterward included into the training data set. In the following subsections, we describe the data set, predictive modeling with supervised models, use of semisupervised learning and virtual patient data, and generation of prediction explanations.

### Data Set

**Figure 2:**
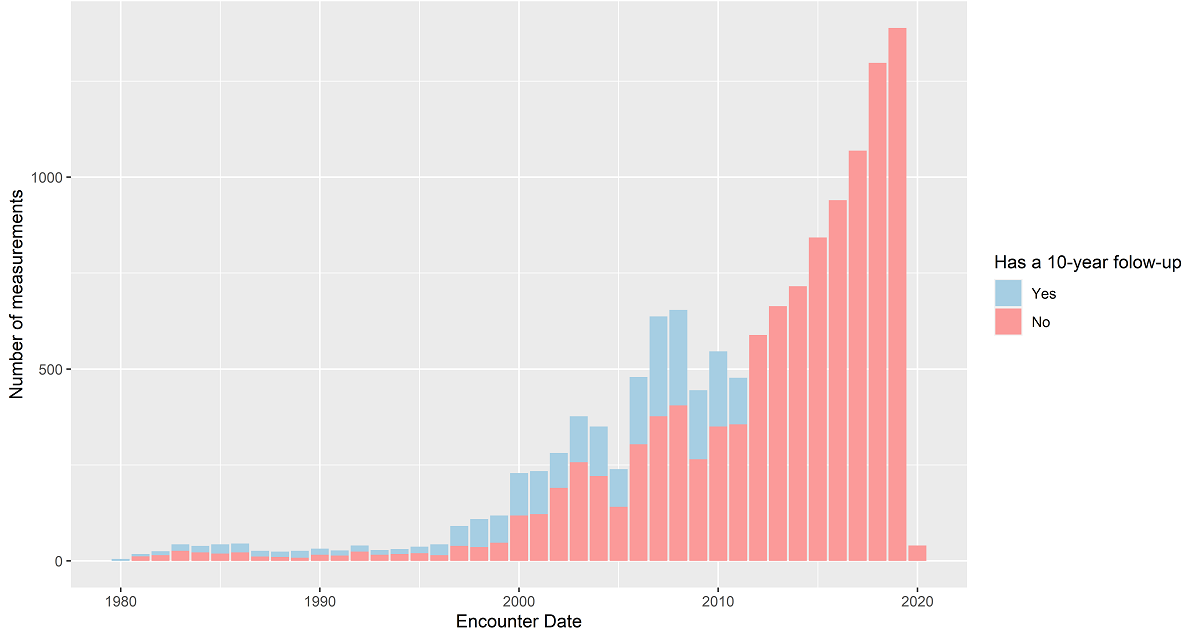
Relationship between the amount of labeled and unlabeled data. The bars for Yes and No values are stacked, visually revealing the ratio between labeled and unlabeled data. Note that the rightmost columns do not have 10-year follow-up data, as they are less than 10 years.

The proposed approach was developed on a data set that was provided by the University of Florence as a result of its long-term clinical practice. The data set included patients who were enrolled over the past 40 years (Figure 2), and 1860 (80.24%) of 2318 patients had at least 1 available 10-year follow-up. They were followed for an average duration of about 7 years and ranging up to 37 years. The data set contains longitudinal clinical data for 2318 patients diagnosed with HCM or patients that had a relative diagnosed with HCM (1457 [62.86%] male and 861 [37.14%] female patients). During the patients' visits, various clinical tests and relevant disease-related events were recorded. These data included general data (gender, age, height, weight, etc), genetic data (detected mutations), clinical tests (echocardiogram [echo], Holter monitoring, blood test, CMR, stress test), prescribed medications (type, start date, termination date), and disease-related events (eg, SCD, heart failure, transplant, abnormal Holter, pacemaker or implantable cardioverter defibrillator implantation). Echo was the leading diagnostic reference technique that was performed for the vast majority of patients and thus represents the main source of data. CMR was additionally used selectively due to its greater accuracy in measuring volumes. Although echo and CMR are treated separately and never computationally compared to each other in medical practice, we used CMR, where available, as an additional data modality to possibly improve prediction accuracy. In total, there were 6227 events recorded, of which 4902 (78.72%) events occurred in patients who were primarily diagnosed with HCM. The structure of the data set therefore allowed observing how patients’ clinical characteristics change over time, which is essential for the desired modeling of HCM progression. The basic patient characteristics are shown in Table 1 for continuous parameters, Table 2 for binary parameters, and Table 3 for the remaining parameters. The characteristics were extracted from 10,318 measurements in total. Additionally, Table 4 shows the missing data numbers and percentages for the 6 selected target variables for their role as input or target variables.

**Table 1:** Basic characteristics of patients for basic continuous parameters (N=10,318).

Continuous parameter	Mean (SD)	Missing data, n (%)
Age (years)	52.1 (18.6)	4 (0.04)
Weight (kg)	73.4 (14.6)	2381 (23.08)
Height (cm)	169 (10.3)	2273 (22.03)
Body mass index (BMI)	25.6 (4.09)	2423 (23.48)
NYHA^a^	1.69 (0.73)	983 (9.53)

^a^NYHA: New York Heart Association.

**Table 2:** Basic characteristics of patients for basic binary parameters (N=10,318).

Binary parameter	1-value, n (%)	0-value, n (%)	Missing, n (%)
Alcohol	Yes, 103 (0.99)	No, 10,215 (99)	0
Drug	Yes, 18 (0.17)	No, 10,300 (99.83)	0
Smoking	Yes, 3437 (33.31)	No, 6881 (66.69)	0
Pregnancy	Yes, 443 (4.29)	No, 9875 (95.71)	2515 (24.37)
Gender	Male, 6400 (62.03)	Female, 3918 (37.97)	0

**Table 3:** Basic characteristics for groups of parameters (N=10,318)^a^.

Procedure	Parameters, n	Total missing values, n (%)
ECG^b^	9	45,839 (49.36)
Echo^c^	26	98,191 (36.60)
CMR^d^	10	81,174 (78.67)

^a^The table shows aggregated statistics for several parameters obtained from the same procedure. The percentage for each procedure is obtained as follows: [Total missing values/(Parameter × N)] × 100.

^b^ECG: electrocardiogram.

^c^Echo: echocardiogram.

^d^CMR: cardiovascular magnetic resonance.

**Table 4:** Absolute number and percentage of missing values of target variables as class and as input (N=10,318).

	LA_d^a^, n (%)	LVEF^b^, n (%)	NYHA^c^, n (%)	LVIDd^d^, n (%)	LVIDs^e^, n (%)	LA_Vol^f^, n (%)
Target	8569 (83.05)	8481 (82.19)	8313 (80.57)	8607 (83.42)	9336 (90.48)	8631 (83.65)
Input	2691 (26.08)	2399 (23.25)	983 (9.53)	2517 (24.39)	5329 (51.65)	3680 (35.67)

^a^LA_d: left atrial diameter.

^b^LVEF: left ventricular ejection fraction.

^c^NYHA: New York Heart Association.

^d^LVIDd: left ventricular internal diameter at end diastole.

^e^LVIDs: left ventricular internal diameter at end systole.

^f^LA_Vol: left atrial volume.

First, we transformed the available data set into a suitable form for predicting a 10-year change in relevant parameters using ML. Similarly, in other real-world data sets, most of the clinical tests were missing many patients or measurements were not taken for the whole span of 10 years (Figure 2). To address this issue, we preprocessed the data as follows:

- Formation of training examples: Since not all clinical tests can be conducted on the same day or in the same month, we defined a training example as a set of measurements within a time frame of 1 year. Such time frame corresponds to the annual regular visit period of patients and allows enough time for relevant changes in the observed parameters to become noticeable, as the disease slowly progresses. If the patient had a certain test performed multiple times within this time frame, multiple tests were treated as separate measurements. In case a certain type of test was not performed in the 1-year time frame, the corresponding variables were recorded as missing. Constructing training examples in this way yielded a data set with 13,386 examples, with 3.9 (SD 4.8) examples per patient.
- Imputation of missing data: The missing values in the data set, either because of nonperformed tests or because of erroneous input of data, were imputed by copying the closest past values (sensible because the progression of HCM is slow; used on numerical and categorical attributes), imputing values of a healthy patient (sampled from the normal distribution; used for numerical attributes), or imputing mean values where healthy values were unknown (used on numerical and categorical attributes). Since measurements were not taken at equidistant time intervals, we used linear interpolation for computing equidistant measurement approximations.

We used the formed training examples as input to supervised learning algorithms. Prior to modeling, we evaluated the quality of attributes, which is important for decreasing learning complexity, avoiding overfitting, and, therefore, improving the simplicity and performance of ML methods. To facilitate learning with NNs, we also scaled the values to the interval [0,1] and encoded nominal values using the one-hot encoding method.

We used RReliefF [38], adaptation of the ReliefF feature selection algorithm, for regression problems. RReliefF calculates how well a feature’s values distinguish between distant labels of instances that are close to each other and considers feature interactions. We selected 21 (18.7%) of 112 attributes based on the average rank across all 6 target variables for further supervised learning. Feature scores for 21 selected features are shown in Table 5, along with their average ranks across 6 trained predictive models. After removing highly correlated features (eg, the weight feature that correlates to the body surface area [BSA] and height), the final set of attributes contained all target variables (regardless of their rank) and the best-performing attributes, based on average rank.

**Table 5:** Selected attributes using RReliefF.^a^

Variable^b^		LA_d^c^ score	LVEF^d^ score	NYHA^e^ score	LVIDd^f^ score	LVIDs^g^ score	LA_Vol^h^ score	Average rank
**Anthropometric parameters**								
	*Age*	0.198	0.194	0.166	0.142	0.166	0.158	1.000
	*Gender*	0.051	0.037	0.043	0.055	0.058	0.022	12.500
	*Height*	0.057	0.064	0.045	0.075	0.051	0.029	9.167
	*BSA* ^i^	0.075	0.073	0.053	0.095	0.085	0.045	4.167
**Risk factors**								
	*Smoking*	0.063	0.046	0.052	0.032	0.069	0.082	7.500
	*Presence of hypercholesterolemia*	0.072	0.042	0.052	0.039	0.044	0.056	9.667
	History of syncope	0.026	0.036	0.029	0.022	0.029	0.048	20.000
	*Family history of HCM* ^j^	0.056	0.060	0.061	0.047	0.052	0.066	5.833
	Family history of SCD^k^	0.027	0.051	0.032	0.031	0.051	0.049	14.667
**Clinical, ECG^l^, and echo^m^ parameters**								
	NYHA	0.011	0.017	0.069	0.007	0.027	0.022	33.000
	Presence of atrial fibrillation	0.055	0.036	0.048	0.018	0.026	0.068	16.333
	QRS duration	0.035	0.046	0.029	0.039	0.026	0.039	17.167
	*Interventricular septum (* *IVS)*	0.043	0.052	0.049	0.041	0.057	0.052	8.167
	LA_d	0.078	0.037	0.036	0.018	0.031	0.070	15.000
	LA_Vol	0.055	0.029	0.026	0.012	0.025	0.059	24.000
	LVIDs	0.017	0.022	0.027	0.029	0.043	0.031	25.167
	LVIDd	0.021	0.017	0.017	0.036	0.044	0.026	27.667
	LVEF	0.018	0.051	0.019	0.014	0.050	0.013	27.833
**Genetics**								
	*Mutation MYBPC3*	0.045	0.041	0.039	0.051	0.052	0.059	9.667
	*Mutation MYH7*	0.037	0.044	0.034	0.040	0.066	0.023	14.667
	Negative genetics	0.036	0.037	0.027	0.043	0.030	0.031	18.667

^a^The table shows RReliefF feature scores and the average ranks for each target variable.

^b^Names of the 10 highest-ranked variables are italicized.

^c^LA_d: left atrial diameter.

^d^LVEF: left ventricular ejection fraction.

^e^NYHA: New York Heart Association.

^f^LVIDd: left ventricular internal diameter at end diastole.

^g^LVIDs: left ventricular internal diameter at end systole.

^h^LA_Vol: left atrial volume.

^i^BSA: body surface area.

^j^HCM: hypertrophic cardiomyopathy.

^k^SCD: sudden cardiac death.

^l^ECG: electrocardiogram.

^m^Echo: echocardiogram.

### Predictive Modeling With Supervised and Semisupervised Machine Learning

To model the relationship between input patient data and target variables, we applied the following supervised learning algorithms:

- RFs [39,40] are an ensemble prediction model that construct multiple randomized decision trees. The implementations of an RF classifier in the R statistical package (library ranger) and the Python Scikit-Lean package [41] were used. Each forest used between 500 and 1500 trees, and the Gini index was used as the attribute-splitting rule.
- Gradient boosting (XGBoost) [42] is an ensemble of weak decision tree predictors, implemented in the open source software library XGBoost.
- LR is a traditional method of finding a linear dependence between attributes and the selected target variable.
- NNs mimic the architecture and working of brain neurons. We used 1 input and 1 output layer and 1 or several hidden layers. In the optimization process, we optimized several learning parameters, such as the learning rate, number of hidden layers, sizes of layers, regularization, sample weights, class weights, dropout, and batch normalization.

The best hyperparameters of these algorithms were tuned using Bayesian optimization and random search implemented in *keras-tuner* [43].

### Semisupervised Learning and Virtual Patients

Semisupervised learning is increasingly used in medicine, especially for medical image segmentation [44-46]. This approach allows labeling a large amount of unlabeled data using only a small portion of labeled data. The majority (ie, 83.9% averaged over 6 target variables) of patients' data did not have records for the follow-up after 10 years. These unlabeled data were used as examples for semisupervised learning, producing a teacher model. The unlabeled examples were labeled with the supervised learning predictive model (see the Predictive Modeling With Supervised and Semisupervised Machine Learning section) and added to the training set. After that, a new model (also called a student model) was trained and kept if it achieved better performance on the test set than the teacher model.

To further improve the results of semisupervised learning, we used artificially generated data (ie, virtual patients). Virtual data generation can sometimes replace experiments in biomedical experiments on animals [47]. Specifically in cardiovascular modeling, patient-specific virtual patient modeling has recently made major progress in improving diagnoses [48]. We evaluated the performance and appropriateness of several virtual patient data generators for this task, such as the generator based on the multivariate normal and log-normal distribution (MVND and log-MVND) [49], and nonparametric methods using supervised tree ensembles, unsupervised tree ensembles, radial basis function–based NNs [50], and Bayesian networks [51]. As the final data generator, we chose the unsupervised tree ensembles, which exhibited the highest level of agreement between the real and the virtual distributions, computed with the Kolmogorov-Smirnoff goodness-of-fit statistical test [52]. We generated 10,000 virtual patient examples, with 20 most important features, listed in Data Set section.

### Explanation of the Predictive Model

Supervised ML models often exhibit a black-box nature, meaning that they can model data but not provide an explanation for the contained knowledge as well as the reasoning used in predictions. This means that the models lack transparency and interpretability. To address this, explanation methods provide justification for each prediction and assess features with the highest impact [53]. This is important in risk-sensitive ML application areas, such as medicine, where the predictions of ML models need to be understood as they may represent a basis for further medical interventions.

In our work, we applied the SHAP method [14], which is a model-agnostic method, generating an explanation for different ML models in a unified form. The method uses theoretically sound concepts of Shapley values from cooperative game theory for computing contributions of each individual attribute value and of each attribute overall. The generated explanations visualize the most relevant attributes that contribute to higher or lower prediction values. The explanations can be computed either for a single patient’s predictions or summarized over all patients to discover more general relationships between attributes and the model’s predictions.

## Results

### Models’ Comparison

To evaluate and compare the performance of the 6 predictive models, we used stratified 10-fold cross-validation. For each of the 6 predictive problems, 4 different regression models were evaluated (LR, RF, gradient-boosted [GB] trees, and NN). The following parameters were varied in tests:

- Application of semisupervised learning (denoted with S)
- Addition of virtual patients' data into the learning data set (denoted with VP)
- Use of all 112 features (denoted with All) or only a subset of the 21 best features (denoted with Subset)
- Interpolation of data points so that measurements were equidistant (denoted with I)

In all, 28 different combinations of the parameters were used in experiments. Some combinations were omitted due to limitations (eg, VP generators cannot generate data for all 112 attributes, so VP was evaluated only with the subset of attributes) or excessive time complexity (eg, the use of virtual patients with NNs).

#### Performance of Predictive Models

To compare the accuracy of the obtained models, we computed the following 4 metrics: mean absolute error (MAE), root-mean-square error (RMSE), and 2 variations of the relative root-mean-square error (RRMSE_mean_ and RRMSE_const_). The MAE measures the average absolute difference between predicted and true values over all examples in the test set. The RMSE addresses the issue that the squared values of the MSE are hard to interpret. The RRMSE measures the relative ratio between the obtained model and the baseline model. We computed 2 variations of the RRMSE with 2 different baseline models: mean predictor and constant predictor. With the RRMSE_mean_, we compared the performance of the obtained model to the model that returned the mean of the target variable over all patients (mean predictor), while with the RRMSE_const_, we compared the obtained model to the model that assumed that the value of the target variable would remain constant/unchanged over the 10-year period (constant predictor).

We summarized (Table 6) the performance of the best-performing predictive models (RF, LR, GB, NN) and parameters (S, VP, All/Subset) for each target variable. We could see that the top-performing regression models were the RF and the GB tree for all target variables. We achieved the best results by applying semisupervised learning (S) for all target variables and using virtual patients (VP) for 5 of 6 target variables. For all targets, the best results were obtained by learning from a subset of the 21 most important features. The values of both RRMSE metrics revealed that the model performs better than the baseline models (their values are less than 1.0), with the model for the LA_d target achieving the lowest predictive error.

**Figure 3:**
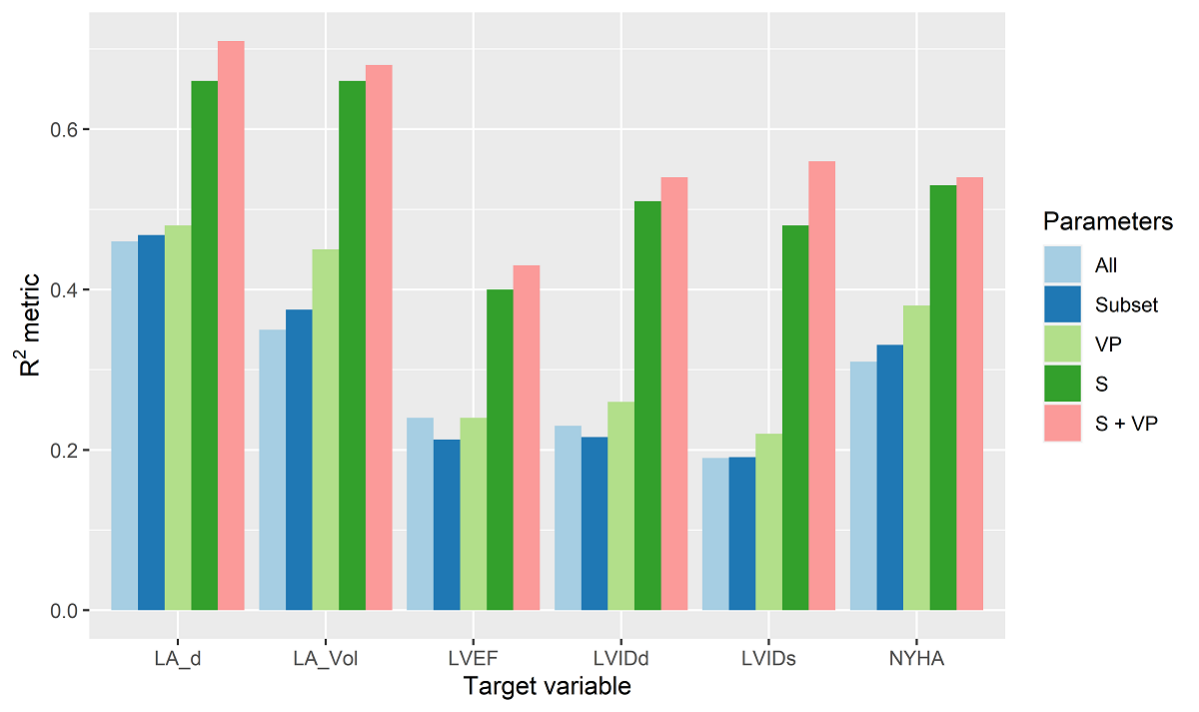
Plotted results for the R^2^ statistic for each target variable using different sets (input parameters). Note that VP, S, and S + VP are used on feature subsets. LA_d: left atrial diameter; LA_Vol: left atrial volume; LVEF: left ventricular ejection fraction; LVIDd: left ventricular internal diameter at end diastole; LVIDs: left ventricular internal diameter at end systole; NYHA: New York Heart Association; S: application of semisupervised learning; VP: addition of virtual patients' data into the learning data set.

To further evaluate the contribution of different data augmentation strategies, we compared the results on different patient sets: original (All features), subset of best features (Subset), virtual patients (VP), semisupervised learning (S), and the combination of the latter 2 (S + VP). The obtained results, shown for the best-performing RF model, are given in Figure 3, which compares the R^2^ metrics for each individual target parameter. The additional detailed results for the other models are given in Multimedia Appendix 1. The obtained results reveal the benefits of reducing the feature space, as well as applying the used data augmentation methods.

In the following subsection, we apply the explanation methodology that helps interpret the computed predictions and their contributing feature values.

**Table 6:** Comparison of the best-performing models for each target variable.

Target	Model and parameter	MAE^a^	RMSE^b^	RRMSE^c^_mean_	RRMSE_const_
LA_d^d^	RF^e^: S^f^+VP^g^+Subset	3.4	4.73	0.54	0.46
LA_Vol^h^	RF: S+VP+Subset	18.4	26.73	0.56	0.47
LVEF^i^	GB^j^: S+Subset	4.92	6.73	0.67	0.61
LVIDd^k^	RF: S+VP+Subset	3.53	5.26	0.68	0.64
LVIDs^l^	RF: S+VP+Subset	3.42	4.81	0.66	0.56
NYHA^m^	RF: S+VP+Subset	0.39	0.5	0.67	0.66

^a^MAE: mean absolute error.

^b^RMSE: root-mean-square error.

^c^RRMSE: relative root-mean-square error.

^d^LA_d: left atrial diameter.

^e^RF: random forest.

^f^S: application of semisupervised learning.

^g^VP: addition of virtual patients' data into the learning data set.

^h^LA_Vol: left atrial volume.

^i^LVEF: left ventricular ejection fraction.

^j^GB: gradient boosted.

^k^LVIDd: left ventricular internal diameter at end diastole.

^l^LVIDs: left ventricular internal diameter at end systole.

^m^NYHA: New York Heart Association.

#### Explanation of Predictions

To augment the output of prediction models, we applied the SHAP method [14] for computing explanations of individual predictions. The explanation of a single prediction consists of relevant textual, graphical, and numerical data that allows understanding of the relationships between the features of the patient and the model’s prediction. It also consists of a list of the most relevant features that influence the prediction, along with their contribution values that define whether the feature value either supports the predicted value or opposes it. The direction of the impact (ie, sign of the contribution value) is denoted using different colors.

**Figure 4:**
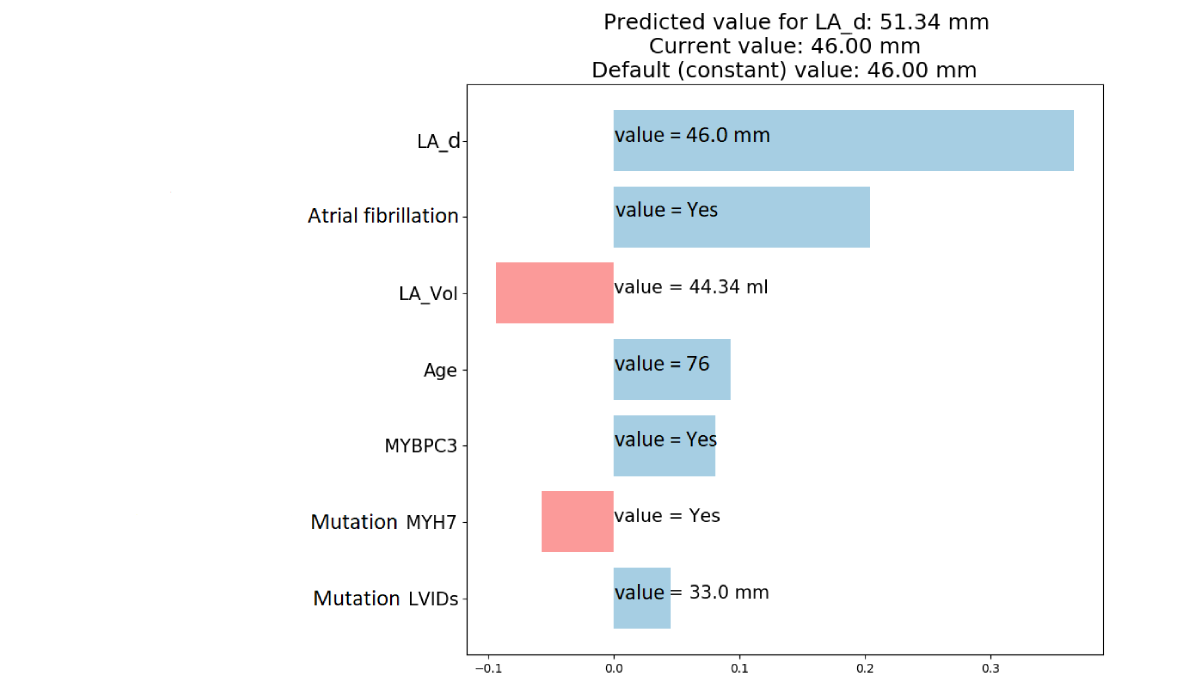
Example of an explanation of the prediction for the target variable LA_d. LA_d: left atrial diameter; LA_Vol: left atrial volume; LVIDs: left ventricular internal diameter at end systole.

An example of the explanation generated for the prediction for the target LA_d (Figure 4) is presented here. Features’ contributions are sorted in descending order, and the graph contains only the features for which the sum of their contributions reflects 95% of the difference between the initial parameter value and the predicted value after 10 years. The green and red bars thus denote positive and negative contributions of the impact for individual feature values, respectively, showing the factors contributing to the increase or decrease in the LA_d value. We can see that the LA_d, atrial fibrillation, age, mutation MYBPC3, and LVIDs features contributed to the increase in the predicted value for LA_d over time, while the LA_Vol and mutation MYH7 features contributed to the decrease in the predicted value for LA_d. Because the overall increasing impact was more prominent, the final predicted value (51.34) was higher than the baseline prediction, which is also the current patients' value of LA_d (46.00). Larger magnitudes of the features' contributions correspond to larger changes in the prediction value. For example, LA_d contributed the most (approximately 30%) to the increase in the predicted value.

### Validation With Medical Experts

Besides evaluation of prediction models with statistical measures conducted in 2 previous sections, we engaged medical experts to provide further interpretation and validation of the results. First, we compared the accuracy of predictive models with the accuracy of human experts, which was obtained by using a survey (Multimedia Appendix 2). Second, we checked whether prediction explanations were sensible and consistent with the experts' medical knowledge about HCM.

We prepared a questionnaire for medical experts and distributed it to several medical universities and cardiology clinics. The questionnaire included data about complete medical cases (measurements, events, and medication data) for 10 patients, and the experts were asked to study them and complete the following 2 tasks:

- Predict the magnitude of the 10-year change in the 6 studied clinical parameters (LA_d, LA_Vol, LVEF, LVIDd, LVIDs, and NYHA) and mark it on a discrete scale from –3 to 3, where –3 and 3 represented the biggest-possible decrease and increase, respectively. Possible magnitudes of change were represented using discrete intervals, as the prediction of an exact value is a difficult task that does not take place in medical practice.
- Evaluate whether the statements generated from the explanation (eg, “The current value of parameter LA_d will cause a decrease in LA_d”) are true or false. For each patient, 6 such statements were generated, covering the features with the highest contribution. More specifically, the questionnaire included evaluation questions for 6 parameters that contribute to a change in LA_d, 4 for LA_Vol, 5 for LVEF, 6 for LVIDd, 7 for LVIDs, and 4 for NYHA.

The questionnaire was fully completed by 13 experts with 16 (SD 8) years of experience. In the following subsections, we present the analysis of the answers.

#### Validation of Prediction Accuracy

To compare the prediction accuracy between the experts and the ML model, we first discretized the model's predictions into discrete intervals so that they could be compared to the discrete intervals, predicted by the experts. We performed the discretization using bins of width 0*.*25*σ*, where *σ* is the SD of the variable. Further, we calculated the following prediction errors:

- Mean prediction error of the discretized model prediction (denoted with MD)
- Mean prediction error made by individual medical experts (denoted with E)
- Mean prediction error of the consortium prediction (ie, the average prediction of all doctors, denoted with C

We could see that the mean prediction error of the discretized model MD (Table 7) was the lowest for all target variables except for LA_d. The mean errors of consortium predictions C were lower than the predictions of individual experts for all parameters, which indicates that the mutual consolidation of different doctors' opinions reduced the error of their joint predictions. The consortium prediction error also turned out to be the lowest for the parameter LA_d and thus better than the error of the ML model.

**Table 7:** Mean absolute error (MAE) of the discretized model predictions (MD), individual experts (E), and the entire consortium (C).

Target/prediction	Model (MD), MAE (SD)	Expert (E), MAE (SD)	Consortium (C), MAE (SD)
NYHA^a^	*0.30* *(0.48)* ^b^	0.84 (0.69)	0.56 (0.34)
LA_d ^c^	1.70 (0.82)	1.69 (0.97)	*1.66* (*0.70*)^b^
LA_Vol^d^	*1.00* *(0.82)* ^b^	1.25 (0.98)	1.13 (0.63)
LVIDd^e^	*0.80* *(0.63)* ^b^	1.09 (0.91)	1.00 (0.77)
LVIDs^f^	*0.50* *(0.71)* ^b^	1.02 (0.86)	0.88 (0.68)
LVEF^g^	*0.90* *(0.88)* ^b^	1.32 (0.90)	1.28 (0.79)

^a^NYHA: New York Heart Association.

^b^The lowest achieved errors are italicized.

^c^LA_d: left atrial diameter.

^d^LA_Vol: left atrial volume.

^e^LVIDd: left ventricular internal diameter at end diastole.

^f^LVIDs: left ventricular internal diameter at end systole.

^g^LVEF: left ventricular ejection fraction.

#### Validation of the Model Explanation

To validate the generated model explanations, we analyzed the agreement of experts with statements generated about the features' influence in 2 steps. First, we calculated the agreement ratio for individual features that were included in the questionnaire, grouped by each of the 6 target variables. Second, we calculated the overall agreement of experts with the explanation for each of the 6 target parameters, based on the agreement data about all features that contributed to their prediction.

The results (Table 8) of the analysis provided the ratio of agreement between different parameters for each target variable, as well as their overall agreement. The highest agreement ratio was achieved for target attributes NYHA (1.00), LA_Vol (0.75), and LVIDd (0.67). The last column (Average agreement) summarizes the results across all used features. The results, in decreasing order, of the last column show that the majority of the experts agreed, especially with the explanations for the targets NYHA (average agreement of 0.73) and LVIDd (average agreement of 0.52). By comparing Tables 7 and 8, we consistently see that the experts least agreed with explanations for the target LA_d, for which the predictive model achieved a larger error than individual experts or the entire consortium. In cases where the predictive model achieved better predictive accuracy than the experts (Table 7) and the agreement of the experts with the explanation was lower (Table 8), for example, for LVEF, LA_Vol, and LVIDs, there are 3 possible explanations:

- The generated explanation might, indeed, provide incorrect information.
- The generated explanation might explain novel relationships between features and target parameters that have not been observed or documented so far.
- It was hard for the experts to evaluate the claims in the questionnaire about the influence of particular features, as these tasks deviate from the established medical practice and require the experts to rely on their subjective experience.

For establishing the reasons for imperfect agreement between the explanation and the experts, further investigation is therefore required. We can conclude that the results provide some evidence that the generated prediction explanation might provide a complementary view at the prediction of HCM-related parameters. Such explanations might represent a tool that the experts could consult while making their decisions.

**Table 8:** Agreement ratios between experts and prediction explanations for parameters that contribute to predicting each target variable. The last two columns provide summary statistics.

Target variable and parameters		Expert agreement	Summary	
			Ratio of agreed features from at least 50% of experts, n	Average agreement, n
**NYHA^a^**				
	*LA_d* ^b^	*0.77* ^c^		
	*Age*	*0.77* ^c^	1.00 (4/4)	0.73
	*LA_Vol* ^d^	*0.62* ^c^		
	*Atrial fibrillation*	*0.77* ^c^		
**LVIDd^e^**				
	BSA^f^	0.15		
	*Gender*	*0.85* ^c^		
	*LVIDd*	*0.65* ^c^	0.67 (4/6)	0.52
	*QRS duration*	*0.69* ^c^		
	LVEF^g^	0.23		
	*Mutation MYH7*	*0.54* ^c^		
**LVEF**				
	QRS duration	0.38		
	*Presence of hypercholesterolemia*	*0.54* ^c^		
	Syncope	0.46	0.40 (2/5)	0.49
	*Gene_Testing_Performed*	*0.69* ^c^		
	NYHA	0.38		
**LA_Vol**				
	*LA_Vol*	*0.69* ^c^		
	*BSA*	*0.54* ^c^	0.75 (3/4)	0.48
	Age	0.15		
	*Atrial fibrillation*	*0.54* ^c^		
**LVIDs**				
	LA_d	0.38		
	LVIDd	0.38		
	*LA_Vol*	*0.62* ^c^		
	*BSA*	*0.85* ^c^	0.43 (3/7)	0.47
	*Mutation MYBPC3*	*0.62* ^c^		
	Interventricular septum (IVS)	0.38		
	Family history of HCM^h^	0.08		
**LA** **_d**				
	*LA_d*	*0.85* ^c^		
	Atrial fibrillation	0.15		
	BSA	0.08	0.17 (1/6)	0.36
	IVS	0.38		
	Age	0.31		
	LVEF	0.38		

^a^NYHA: New York Heart Association.

^b^LA_d: left atrial diameter.

^c^Names of parameters with agreement higher than 50% are italicized.

^d^LA_Vol: left atrial volume.

^e^LVIDd: left ventricular internal diameter at end diastole.

^f^BSA: body surface area.

^g^LVEF: left ventricular ejection fraction.

^i^HCM: hypertrophic cardiomyopathy.

## Discussion

### Principal Results

We presented a disease progression system for patients diagnosed with HCM that is based on predicting 6 target parameters (LA_d, LA_Vol, LVIDd, LVIDs, LVEF, and NYHA) for 10 years ahead using supervised ML models. The experiments revealed good ML performance for all targets, with the achieved predictive error lower than the error of the default predictors. The experiments also revealed that semisupervised learning and the artificial data from virtual patients helped achieve even higher predictive accuracy for all 6 targets. Finally, we validated our approach with human experts using a structured questionnaire and determined the models' favorable performance compared to performance of experts for 5 of 6 targets.

### Limitations

The design of the study carried several limitations, stemming from the fact that this work was based on real-world data that are expensive to obtain and are subject to noise. The first limitation of this study is that it was based only on a single medical center data set. To further validate this study, it would be beneficial to independently evaluate the models with data sets from other centers or extend the existing data set with more data. Additionally, the benefit for including more data could also be in diminishing a potential bias of our data set, which could potentially include a population distribution that is different from other medical centers and thus different ranges of recorded parameters, which we did, in fact, observe in some cases. Additionally, in the perfect but rather unrealistic scenario due to its cost, both data modalities (echo and CMR) would be available for all patients, which would allow us to use the CMR data as an additional data source for all patients. Due to the unavailability of such data at the time of the study or data that were structured differently, we leave this for our further work.

Further, to prepare the data to be used for ML and obtain stable predictions, we used several preprocessing and data augmentation steps. Since we are dealing with real medical data, this opens questions of how different data transformations influence our predictions. Hence, a sensitivity study of the results would be required, as well as determining how the patient’s record time frame and predicted risk time frame influence the achieved accuracies. An additional limitation of the performed validation was that the ML results were compared to the inputs of medical experts in the structured survey instead of their free diagnoses and evaluations. Although this was required to unify the structure of human answers to enable statistical comparisons, the form of survey might introduce its own bias.

The described limitations, along with our further research questions and ideas, open several ideas for future study directions. First, we will evaluate the proposed system on an independent cardiological data set (eg, the Sarcomeric Human Cardiomyopathy Registry [SHaRe]) [54]. Second, as our current approach provides future predictions for 6 independent parameters, the outputs will be further combined into a single risk prediction of high/low risk, which can further improve HCM health management initiative [32]. To achieve this, a combination of models' output analysis and domain experts' input would be required. Finally, further ways for improvement of predictive accuracy will be tested (additional predictive models and feature selection techniques, including deep learning), as well as determining the reasons for the experts' disagreement with some of the explanation components.

### Conclusion

Although ML can have limitations in medicine [2], in this work, we showed the importance of using computer models in cardiology by predicting disease progression of HCM patients 10 years ahead, which could be used to prevent SCD. Additionally, the results confirmed findings in Chen et al [44], Gu et al [45], and Bai et al [46] that additional artificial data and semisupervised learning can provide additional low-cost and low-risk data using already available medical knowledge, increasing the predictive performance. Simple explanations of predictions contribute to the trust of provided predictions and ease the decision of experts. We hope that our work will further contribute to the goal of developing constructive strategies to prevent SCD in patients with HCM, as motivated by Maron et al [36].

## Appendix

Multimedia Appendix 1 Bar graphs of parameter influence for each model used.

Multimedia Appendix 2 A sample of the questionnaire for the first patient.

## Abbreviations

AI: artificial intelligence
BSA: body surface area
CMR: cardiac magnetic resonance
COPD: chronic obstructive pulmonary disease
ECG: electrocardiogram
Echo: echocardiogram
GB: gradient boosted
HCM: hypertrophic cardiomyopathy
KNN: k-nearest neighbors
LA_d: left atrial diameter
LA_Vol: left atrial volume
LR: linear regression
LVEF: left ventricular ejection fraction LVIDd: left ventricular internal diameter at end diastole
LVIDs: left ventricular internal diameter at end systole
MAE: mean absolute error
ML: machine learning
MVND: multivariate normal distribution
NN: neural network
NYHA: New York Heart Association
RF: random forest
RMSE: root-mean-square error
RRMSE: relative root-mean-square error
SCD: sudden cardiac death
SHAP: Shapley additive explanation
SVM: support vector machine
