# Supplementary figures and images for: Disease Progression of Hypertrophic Cardiomyopathy: Modeling Using Machine Learning
Source: JMIR Med Inform. 2022 Feb 2;10(2):e30483. doi: 10.2196/30483 (PMC8851344; doi:10.2196/30483)

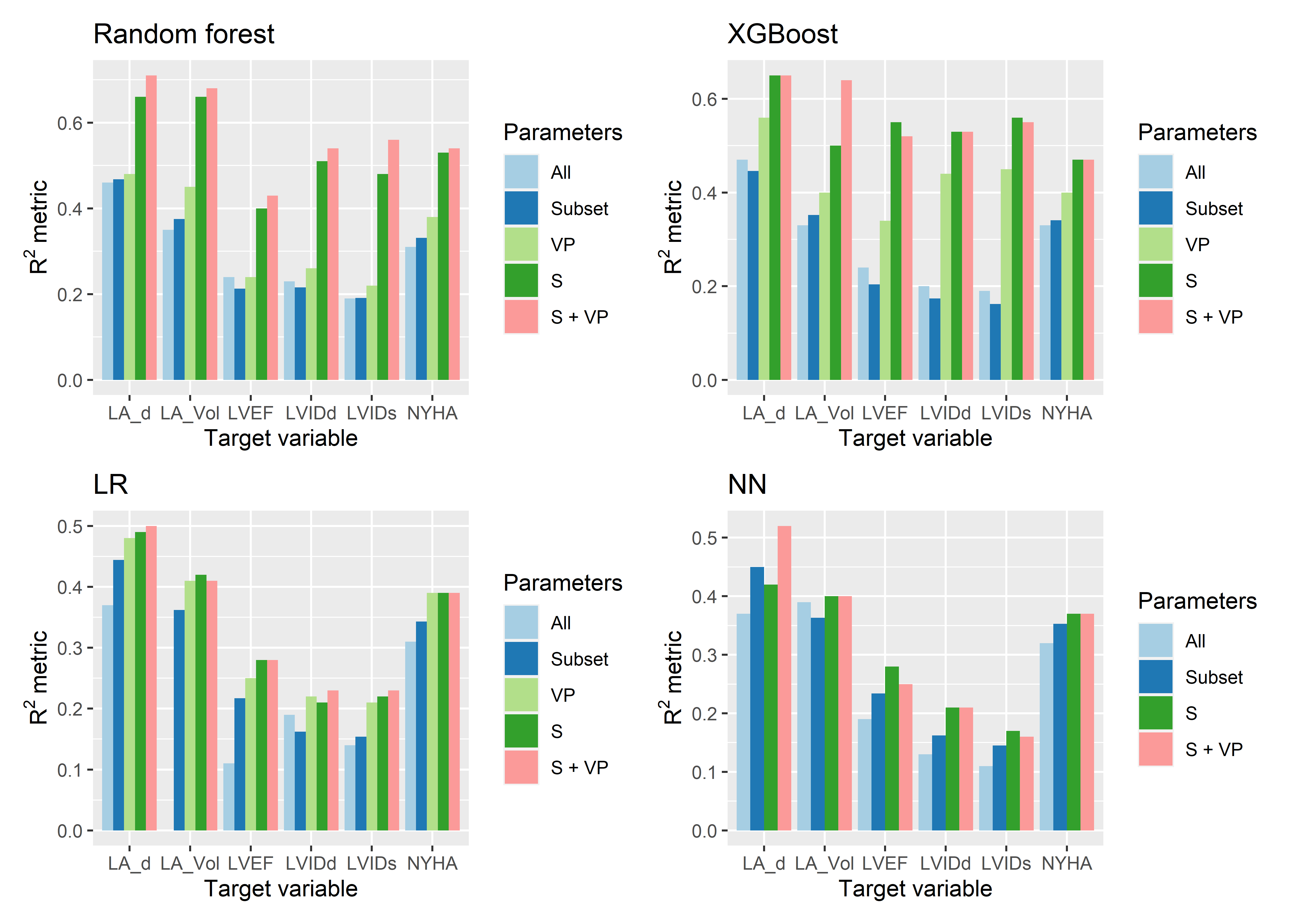

Supplement: Multimedia Appendix 1 [file medinform_v10i2e30483_app1.png]
