# Supplementary material for: Disease Progression of Hypertrophic Cardiomyopathy: Modeling Using Machine Learning
Source: JMIR Med Inform. 2022 Feb 2;10(2):e30483. doi: 10.2196/30483 (PMC8851344; doi:10.2196/30483)
Supplement: Multimedia Appendix 2 [file medinform_v10i2e30483_app2.pdf]

Preview - data is not collected.

Correlation between expert and disease-progression model predictions

Patient summary - patient 1

The following tables are a summary of relevant patient data. If you feel that you need additional information the full patient data is provided at the end of this page.

`Measurements:`

Encounter_Date	03.07.2017	08.08.2017	02.10.2017	19.10.2017	28.12.2017	30.08.2018	27.12.2018	08.01.2020	20.07.2020
Age	31.390104	31.488668	31.639253	31.685798	31.877451	32.548238	32.874049	33.906240	34.437394
Gender	MALE	MALE	MALE	MALE	MALE	MALE	MALE	MALE	MALE
BMI	25.6	25.6	25.6	25.6	25.3	25.3	25.3	26.0	30.1
NYHA	3.0	3.0	2.0	1.0	1.0		1.0	1.0	2.0
FHx_HCM	0.0	0.0	0.0	0.0	0.0	0.0	0.0	0.0	0.0
FHx_SCD	0.0	0.0	0.0	0.0	0.0	0.0	0.0	0.0	0.0
Diagnosis__DIABETES	0.0	0.0	0.0	0.0	0.0	0.0	0.0	0.0	0.0
Diagnosis__HYPERTENSION	0.0	0.0	0.0	0.0	0.0	0.0	0.0	0.0	0.0
Gene_Test_Negative	1.0	1.0	1.0	1.0	1.0	1.0	1.0	1.0	1.0
ECG_Inverted_T_Waves	0.0	0.0	0.0	0.0	0.0	0.0	0.0	0.0	0.0
ECG_Pathological_Q_Waves	0.0	0.0	0.0	0.0	0.0	0.0	0.0	0.0	0.0
ECG_PR				120.0	120.0	122.0	126.0		
ECG_QRS				138.0	138.0		124.0		
ECG_QTc				459.0	459.0	465.0	443.0		
ECG_Rhythm	Sinus Rhythm	Sinus Rhythm	Sinus Rhythm	Sinus Rhythm	Sinus Rhythm	Sinus Rhythm	Sinus Rhythm	Sinus Rhythm	Sinus Rhythm

Preview - data is not collected.

Ech_Echo_PW	13.0	10.0		12.0	14.0	11.0	11.0	12.0	10.0
Ech_Echo_LA	43.0	36.0	44.0	44.0	43.0	34.0	37.0		42.0
Ech_Echo_LA_Vol	60.0	96.0	75.0	88.0	89.0	85.0	89.0	78.0	75.0
Ech_Echo_LVEF	62.0	66.0	60.0	67.0	68.0	83.0	75.0	67.0	63.0
Ech_Echo_Max_LVT	26.0	22.0	28.0	15.0	15.0	12.0	13.0	13.0	13.0
Ech_Echo_LVIDd	42.0	44.0	49.0	49.0	49.0	55.0	54.0	57.0	52.0
Ech_Echo_LVIDs	21.0		32.0	28.0	20.0	24.0	26.0	24.0	27.0
Ech_Mitral_Regurg_Grade					2.0	1.0			
Ech_Tricuspid_Regurg_Grade									
Ech_doppler_LVOTO_Provocation	5.0								
Ech_doppler_LVOTO_Rest	5.0	50.0	22.0	6.0	13.0		60.0		53.0
Ech_doppler_Mitral_Valve_E_DT		320.0	190.0	180.0	196.0	205.0	204.0	188.0	
CRE_Value									
HGB_Value									
NTBNP_Value								187.4	187.4
CMR_CMR_LV_Mass	236.0	236.0	236.0	239.0	239.0	239.0	239.0		
CMR_GD_Del_Enhance	1.0	1.0	1.0	0.0	0.0	0.0	0.0	0.0	0.0
CMR_CMR_LVSV	93.0	93.0	93.0	102.2	102.2	102.2	102.2		
CMR_CMR_RVSV									

Events:

Diagnosis_Date	Diagnosis
24.09.2017	MYECTOMY

Medications:

Med_Name	Med_Start_Date	Med_Stop_Date
NADOLOLO	09.08.2017	04.09.2018

Task #1 - diagnostic output

Preview - data is not collected.

Based on the current clinical picture and past data (in Patient summary), predict the change (**increase**/**decrease**/**no change**) of the following 6 clinical parameters after 10 years: LA, LA_Vol, LVEF, LVIDd, LVIDs, NYHA. The scale (from -3 to +3) represents the magnitude of the decrease (-3 stands for a big **decrease** and -1 for a small one) or increase (+1 stands for a small **increase** and +3 for a big one), and 0 means there will be **no change.**

	-3	-2	-1	0	1	2	3
Target parameter: LA	☐	☐	☐	☐	☐	☐	☐
Target parameter: LA vol.	☐	☐	☐	☐	☐	☐	☐
Target parameter: LVEF	☐	☐	☐	☐	☐	☐	☐
Target parameter: LVIDd	☐	☐	☐	☐	☐	☐	☐
Target parameter: LVIDs	☐	☐	☐	☐	☐	☐	☐
Target parameter: NYHA	☐	☐	☐	☐	☐	☐	☐

Task #2 - confidence in predictions

Based on the predicted change in the 6 clinical parameters after 10 years in Task #1, estimate **how confident you are** in your prediction. The scale (from 0 to +6) represents the magnitude of your confidence for determining the prediction. Value 0 stands for a very low confidence in prediction, 3 for fairly confident prediction and 6 for a very confident prediction.

	0	1	2	3	4	5	6
Target parameter: LA	☐	☐	☐	☐	☐	☐	☐
Target parameter: LA vol.	☐	☐	☐	☐	☐	☐	☐
Target parameter: LVEF	☐	☐	☐	☐	☐	☐	☐
Target parameter: LVIDd	☐	☐	☐	☐	☐	☐	☐

Preview - data is not collected.

Target parameter: LVIDs	☐	☐	☐	☐	☐	☐	☐
Target parameter: NYHA	☐	☐	☐	☐	☐	☐	☐

Task #3 - status in 10 years

Based on the current clinical picture and past data (in the previous section), predict the patients' status: in which of the three branches (stable, adverse remodeling, overt dysfunction) will each patient be in 10 years.

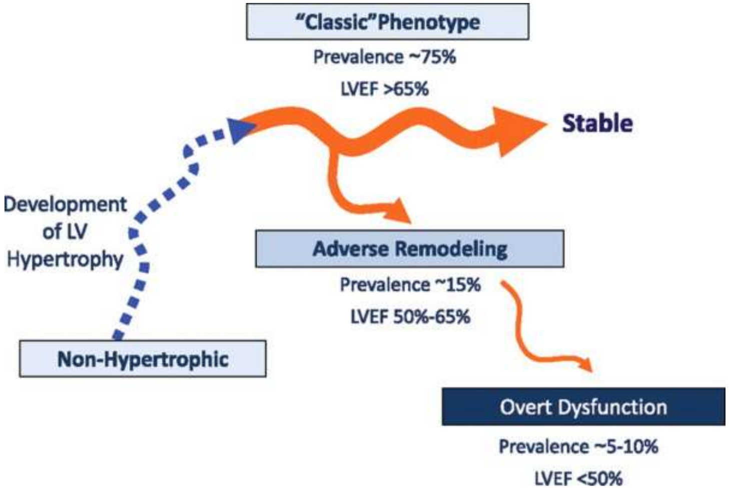

- ☐ Stable
- ☐ Adverse remodeling
- ☐ Overt dysfunction

Preview - data is not collected.

Task #4 - prediction explanation

Determine whether clinical parameters in question increase or decrease the predicted value for target parameters (LA, LVEF, LVIDd, LVIDs, NYHA, LA_Vol) in 10 years after the current state.

1. The current value of the clinical parameter *Ech_Echo_LA* will cause a *decrease* of the predicted value of the target parameter *LA*.

- ☐ True
- ☐ False

2. The current value of the clinical parameter *ECG_QRS* will cause an *increase* of the predicted value of the target parameter *LVEF*.

- ☐ True
- ☐ False

3. The current value of the clinical parameter *Ech_Echo_LVIDd* will cause an *increase* of the predicted value of the target parameter *LVIDs*.

- ☐ True
- ☐ False

4. The current value of the clinical parameter *Age* will cause an *increase* of the predicted value of the target parameter *NYHA*.

- ☐ True
- ☐ False

Preview - data is not collected.

Optional supplement - Full patient data

Measurements:

Encounter_Date	Age	Gender	Weight	Height	BMI	BSA	Primary_Diagnosis	NYHA	FHx_DCM	FHx_HCM	FHx_SCD	FHx_CAD	Diagnosis__DIABETES	Dia
03.07.2017	31.390104	MALE	74.0	170.0	25.6	1.87	HCM	3.0	0.0	0.0	0.0	0.0	0.0	0.0
08.08.2017	31.488668	MALE	74.0	170.0	25.6	1.87	HCM	3.0	0.0	0.0	0.0	0.0	0.0	0.0
02.10.2017	31.639253	MALE	74.0	170.0	25.6	1.87	HCM	2.0	0.0	0.0	0.0	0.0	0.0	0.0
19.10.2017	31.685798	MALE	74.0	170.0	25.6	1.87	HCM	1.0	0.0	0.0	0.0	0.0	0.0	0.0
28.12.2017	31.877451	MALE	73.0	170.0	25.3	1.86	HCM	1.0	0.0	0.0	0.0	0.0	0.0	0.0
30.08.2018	32.548238	MALE	73.0	170.0	25.3	1.86	HCM		0.0	0.0	0.0	0.0	0.0	0.0
27.12.2018	32.874049	MALE	73.0	170.0	25.3	1.86	HCM	1.0	0.0	0.0	0.0	0.0	0.0	0.0
08.01.2020	33.906240	MALE	75.0	170.0	26.0	1.88	HCM	1.0	0.0	0.0	0.0	0.0	0.0	0.0
20.07.2020	34.437394	MALE	87.0	170.0	30.1	2.03	HCM	2.0	0.0	0.0	0.0	0.0	0.0	0.0

Events:

Diagnosis_Date	Diagnosis
24.09.2017	MYECTOMY

Medications:

Med_Name	Med_Start_Date	Med_Stop_Date

Preview - data is not collected.

NADOLOLO	09.08.2017	04.09.2018

Previous page

Next page
